# Prostaglandin E_2_ Exerts Multiple Regulatory Actions on Human Obese Adipose Tissue Remodeling, Inflammation, Adaptive Thermogenesis and Lipolysis

**DOI:** 10.1371/journal.pone.0153751

**Published:** 2016-04-28

**Authors:** Verónica García-Alonso, Esther Titos, Jose Alcaraz-Quiles, Bibiana Rius, Aritz Lopategi, Cristina López-Vicario, Per-Johan Jakobsson, Salvadora Delgado, Juanjo Lozano, Joan Clària

**Affiliations:** 1 Department of Biochemistry and Molecular Genetics, Hospital Clínic-IDIBAPS, Barcelona, Spain; 2 CIBERehd, Hospital Clínic-IDIBAPS, Barcelona, Spain; 3 Department of Medicine, Karolinska Institutet, Stockholm, Sweden; 4 Department of Gastrointestinal Surgery, Hospital Clínic-IDIBAPS, Barcelona, Spain; 5 Department of Biomedicine, University of Barcelona, Barcelona, Spain; Fundação Oswaldo Cruz, BRAZIL

## Abstract

Obesity induces white adipose tissue (WAT) dysfunction characterized by unremitting inflammation and fibrosis, impaired adaptive thermogenesis and increased lipolysis. Prostaglandins (PGs) are powerful lipid mediators that influence the homeostasis of several organs and tissues. The aim of the current study was to explore the regulatory actions of PGs in human omental WAT collected from obese patients undergoing laparoscopic bariatric surgery. In addition to adipocyte hypertrophy, obese WAT showed remarkable inflammation and total and pericellular fibrosis. In this tissue, a unique molecular signature characterized by altered expression of genes involved in inflammation, fibrosis and WAT browning was identified by microarray analysis. Targeted LC-MS/MS lipidomic analysis identified increased PGE_2_ levels in obese fat in the context of a remarkable COX-2 induction and in the absence of changes in the expression of terminal prostaglandin E synthases (i.e. mPGES-1, mPGES-2 and cPGES). IPA analysis established PGE_2_ as a common top regulator of the fibrogenic/inflammatory process present in this tissue. Exogenous addition of PGE_2_ significantly reduced the expression of fibrogenic genes in human WAT explants and significantly down-regulated Col1α1, Col1α2 and αSMA in differentiated 3T3 adipocytes exposed to TGF-β. In addition, PGE_2_ inhibited the expression of inflammatory genes (i.e. IL-6 and MCP-1) in WAT explants as well as in adipocytes challenged with LPS. PGE_2_ anti-inflammatory actions were confirmed by microarray analysis of human pre-adipocytes incubated with this prostanoid. Moreover, PGE_2_ induced expression of brown markers (UCP1 and PRDM16) in WAT and adipocytes, but not in pre-adipocytes, suggesting that PGE_2_ might induce the trans-differentiation of adipocytes towards beige/brite cells. Finally, PGE_2_ inhibited isoproterenol-induced adipocyte lipolysis. Taken together, these findings identify PGE_2_ as a regulator of the complex network of interactions driving uncontrolled inflammation and fibrosis and impaired adaptive thermogenesis and lipolysis in human obese visceral WAT.

## Introduction

Prostaglandin (PG) E_2_ is one of the most abundant lipid mediators in the human body. It is constitutively produced in nearly all tissues by the coordinate enzymatic activities of cyclooxygenases (COX) and terminal PGE synthases [1–4]. PGE_2_ is a powerful molecule that exerts multiple biological effects depending on the tissue environment and the cell type [1–4]. In this regard, in addition to being recognized as an important mediator of inflammation, pain and fever, PGE_2_ also plays an important role in the regulation of vascular tone and cell proliferation and differentiation [5–7].

White adipose tissue (WAT) is a complex and highly active endocrine organ that plays a key role in the regulation of energy metabolism. In obese individuals, WAT expands its energy-buffering capacity by fat cell hypertrophy and/or by hyperplasia from committed progenitors [8]. This adipose tissue expansion leads to a plethora of functional derangements including hypoxia, lack of nutrients and tissue remodeling, which are major contributors to the chronic “low-grade” state of mild inflammation present in WAT of obese individuals [9–11]. This persistent and unresolved inflammatory state in WAT is, in turn, responsible for the excessive synthesis of extracellular matrix components and the subsequent interstitial deposition of fibrotic material [12,13]. Increased interstitial WAT fibrosis decreases extracellular matrix flexibility and reduces the tissue plasticity, which ultimately leads to adipocyte dysfunction [12]. The ultimate consequence of these derangements is the development of a number of comorbidities associated with obesity including insulin resistance and type 2 diabetes, non-alcoholic fatty liver disease, atherosclerosis and cardiovascular disease [14,15].

We recently described that PGE_2_ participates in the differentiation of WAT pre-adipocytes into beige/brite cells [16]. Since beige/brite cells are able to dissipate large amounts of chemical energy as heat by uncoupling protein 1 (UCP1), which uncouples the synthesis of ATP from the respiratory chain [17], this finding was interpreted as favorable within the context of metabolic homeostasis of obese WAT. The aim of the current study was to translate and expand this finding to human obesity by investigating the potential metabolic benefits of PGE_2_ in WAT remodelling, inflammation, adaptive thermogenesis and lipolysis in omental adipose tissue obtained from obese individuals undergoing bariatric surgery. Our data provide evidence that PGE_2_ exerts pleiotropic regulatory effects in the complex homeostasis of WAT in human obesity.

## Materials and Methods

### Reagents

PGE_2_ was obtained from Cayman Chemicals (Ann Arbor, MI). Krebs-Ringer bicarbonate buffer, Dulbecco's Modified Eagle's Medium (DMEM), fatty acid-free (FAF)-BSA and liberase were from Roche (Basel, Switzerland). Nylon mesh filters (100 μm) were obtained from BD Biosciences (San Jose, CA). TRIzol was from Invitrogen (Carlsbad, CA) and L-Glutamine from Biological Industries (Kibbutz Beit Haemek, Israel). FBS and Dulbecco’s PBS with and without calcium and magnesium (DPBS^+/+^ and DPBS^-/-^, respectively) were obtained from Lonza (Vervieres, Belgium). The free glycerol assay kit was from Sigma (St. Louis, MO). The High-Capacity Archive Kit and TaqMan Gene Expression Assays were provided by Applied Biosystems (Foster City, CA). Adipocyte maintenance medium was from Zen Bio (Research Triangle Park, NC). The Pierce BCA Protein Assay kit was from Thermo Scientific (Carlsbad, CA).

### Study participants and sample collection

Twelve obese individuals undergoing laparoscopic bariatric surgery and 10 patients without a history of obesity undergoing laparoscopic cholecystectomy were recruited at the Gastroenterology Surgery Unit of the Hospital Clínic of Barcelona. The participants were selected according to their body mass index (BMI) calculated as mass/height^2^ and categorized as non-obese control (BMI<29.9 kg/m^2^) and obese (BMI>29.9 kg/m^2^) groups. Individuals with the presence of inflammatory bowel disease or cancer, obese patients with previous bariatric surgical procedures or patients with active prescription for any non-steroidal anti-inflammatory drug (NSAID) or any other medication that could affect PG synthesis were excluded from the study. Demographic and clinical data were collected from the electronic medical records of the patients at the time of surgery. Omental adipose tissue samples were harvested and washed twice with DPBS and minced into approximately 100 mg pieces. Tissue samples were either fixed in 10% formalin or snap-frozen in liquid nitrogen and stored at -80°C for further analysis. Some adipose tissue samples were used *ex vivo* or for the isolation of primary adipocytes and pre-adipocytes. This study was approved by the Investigation and Ethics Committee of the Hospital Clínic and written informed consent was obtained from all the participants (protocol # 12–7239).

### Assessment of adipose tissue fibrosis by Sirius Red and Masson’s trichrome staining

Fibrosis in adipose tissue was assessed in paraffin sections by Sirius Red staining. Briefly, sections were incubated for 10 minutes in 0.5% thiosemicarbazide and stained in 0.1% Sirius Red F3B in saturated picric acid for 1 hour, and subsequently washed with an acetic acid solution (0.5%). Fibrosis was also assessed by Masson’s trichrome staining at the Pathology Department of the Hospital Clínic.

### Assessment of adipose tissue fibrosis by bright field and polarization microscopy

Sections of visceral adipose tissue stained with picrosirius red were visualized in a DMRB-Leica microscope (Wetzlard, Germany), equipped with a wide-field Leica DFC 450 camera and a λ analyzer and polarizer. Acquisition of the images was performed with the LAS 4.0 Leica software at 20xPL FLUOTAR Dry Numerical Aperture 0.50 and 40X PL FLUOTAR 1–0.5 oil. The same region of the sample was imaged using bright field to visualize picrosirius stained tissue and polarized light to observe collagen fibbers. Image analysis was performed using FIJI-Image J (Wayne Rasband, NIH). Briefly, images of bright field and polarized light were background subtracted, filtered and automatically thresholded to segment and quantify areas of cellular membrane in bright field images and areas of fibrotic regions visualized under polarized light. Total fibrosis quantification (sum of collagen I and III fibers) was expressed as the ratio of fibrous regions/cellular membranes as described in reference 13. The same procedure was used for quantification of pericellular fibrosis.

### *Ex vivo* experiments in adipose tissue explants

Human WAT explants collected under sterile conditions were placed in P60 plates in pre-warmed (37°C) DPBS^+/+^ containing antibiotics (100 U/ml penicillin and 100 mg/ml streptomycin). Connective tissue and blood vessels were removed by dissection before cutting the tissue into small pieces. The explants were washed with DPBS at 37°C by centrifugation (400g, 1 min) to remove blood cells and pieces of tissue containing insufficient adipocytes to float. Thereafter, the explants were cultured in DMEM with L-glutamine (2 mM), antibiotics and 10% FAF-BSA. Tissue explants of approximately 40 mg were subsequently cultured in 12-well plates with vehicle (0.4% ethanol) or PGE_2_ (1 μM) in 1% FAF-BSA for 24 h.

### Isolation of adipocytes and pre-adipocytes

WAT was excised, weighed and rinsed twice in cold carbogen-gassed Krebs-Ringer bicarbonate buffer supplemented with 1.5% FAF-BSA and 2 mM EDTA and centrifuged at 800g for 1 min at 4°C to remove free erythrocytes and leukocytes. Tissue suspensions (300–600 mg) were placed in 5 ml of digestion buffer containing Krebs-Ringer supplemented with 1.5% FAF-BSA and 1 mg/ml of liberase and incubated at 37°C for 30 min as described [18]. Floating cells corresponding to adipocytes were collected and cultured in DMEM with FBS (10%), L-glutamine (2 mM), antibiotics and HEPES (20 mM). The remaining supernatant was centrifuged at 800g for 5 min and pelleted cells corresponding to the stromal vascular cell (SVC) fraction were incubated with erythrocyte lysis buffer (0.15 M NH_4_Cl, 10 mM KHCO_3_, and 0.1 mM EDTA) for 5 min and centrifuged at 500g for 5 min, as described (16). Thereafter, SVC were incubated in a culture flask containing pre-adipocyte priming DMEM/F-12/Ham’s mediun with L-glutamine, HEPES, 10% FBS and antibiotics for 24 hours.

### Pre-adipocyte differentiation into beige/brite cells

Freshly isolated WAT pre-adipocytes were cultured in DMEM containing 3% FBS, 100 nM insulin and antibiotics and incubated with vehicle (0.01% ethanol) or PGE_2_ (0.1 μM) for 3 hours. The induction medium was replaced with fresh adipocyte maintenance medium (33 μM biotin, 17 μM pantothenate, 1 μM dexamethasone) in the presence of 1 nM triiodothyronine (T3) for 2 additional days. Thereafter, the cells were incubated in adipocyte medium until day 10.

### Differentiation of 3T3-L1 adipocytes

Mouse 3T3-L1 cells were seeded onto 24-well plates (75.000 cells/well) with DMEM supplemented with 10% FBS and maintained in a 5% CO_2_ atmosphere. Cells were allowed to grow to confluence for 2 days and then exposed to DMEM containing a differentiation cocktail (5 μg/ml insulin, 0.25 μM dexamethasone and 0.5 mM IBMX) supplemented with antibiotics and 2 mM L-glutamine in the presence of vehicle (0.01% DMSO), PGE_2_ (0.1, 1 and 5 μM) or compound III (1, 5 and 8 μM). After 48 h, the cells were grown in fresh DMEM with 10% FBS and 5 μg/ml insulin. Finally, the medium was replaced by fresh DMEM after 48h. Six days later, the cells were incubated with vehicle or LPS (100 ng/ml) for 6 h. In some experiments cells were allowed to grow to confluence for 2 days and then incubated in DMEM containing a differentiation cocktail in the presence of vehicle (0.01% DMSO), TGF-β (10 ng/ml) and PGE_2_ (0.1, 1 and 5 μM). After 48 h, the cells were grown in fresh DMEM with 10% FBS and 5 μg/ml insulin. Finally, the medium was replaced by fresh DMEM after 48 h.

### High-throughput transcriptomic analysis

Total RNA was obtained from omental WAT collected from obese patients (n = 4) and non-obese individuals (n = 4). RNA was also obtained from primary cultures of human pre-adipocytes (n = 8) incubated in the absence or presence of PGE_2_ (1 μM) for 3 h. Affymetrix Human Genome U219 expression arrays containing more than 36,000 transcripts and variants (Affymetrix, Inc., Santa Clara, CA) were used to process all the samples. The preparation of cRNA probes, hybridization, and scanning of arrays were performed according to the manufacturer’s protocol and carried out at the Functional Genomics Unit of IDIBAPS. Affymetrix gene expression data were normalized with the robust multiarray algorithm using a custom probe set definition that mapped probes directly to Entrez Gene Ids (HGU219_Hs_ENTREZG). A filtering step excluding probes not reaching a coefficient of variation of 0.02 was employed, and the final number of probes reached a total of 13,928 probes. For the detection of differentially expressed genes, a linear model was fitted to the data and empirical Bayes moderated statistics were calculated using the Limma package from Bioconductor (Seattle, WA). Adjustment of p-values was made by the determination of false discovery rates (FDR) using the Benjamini-Hochberg procedure [19]. All computations were made using R statistical software. Genes representing a fold change of 1.5 or greater and a moderate p-value <0.05 were considered as differentially expressed. The array data were analyzed using the IPA Analysis software. The significance of the association between the data set and the canonical pathway was determined based on a *P* value calculated using the Fischer's exact test determining the probability that the association between the genes in the data set and the canonical pathway is due to chance alone. The microarray data have been deposited in Gene Expression Omnibus (GEO): GSE7141.

### Lipidomic analysis of eicosanoids by LC-ESI-MS/MS

COX-derived products were measured in adipose tissue samples (30 mg) from obese and non-obese subjects by targeted LC-ESI-MS/MS analysis. Samples were extracted using solid phase extraction columns and the hexane elute was evaporated before injection into an Agilent 1200 HPLC system with a binary pump, degasser, autosampler and column thermostat with a Kinetex C-18, 2.1 x 150 mm, 2.6 μm (Phenomenex, Aschaffenburg, Germany) column using a solvent system of aqueous formic acid (0.1%) and acetonitrile. The elution gradient was started with 5% acetonitrile, which was increased to 55% within 0.5 minutes, to 69% in 14.5 minutes, and 95% in 14.6 minutes where it was maintained for 5.4 minutes. The flow rate was set at 0.3 mL/min and the injection volume was 7.5 μL. The HPLC was coupled with an Agilent 6460 Triplequad mass spectrometer (Agilent Technologies, Santa Clara, CA) with an electrospray ionisation source. The source parameters were: drying gas: 250°C/10 L/min, sheath gas: 400°C/10 L/min, capillary voltage: 4500 V, nozzel voltage: 1500 V and nebulizer pressure: 30 psi. The LC-MS/MS conditions are given in S1 Table.

### Lipolysis assay

Human adipocytes were harvested and incubated in DMEM containing 10% FBS in the presence or absence of isoproterenol (1 μM) and PGE_2_ (1 μM) for 1.5–3 h at 37°C. Glycerol release into the media was determined using a free-glycerol assay kit. Glycerol levels were normalized to the total protein content of the primary adipocytes using the BCA protein assay kit.

### Analysis of gene expression by real-time PCR

Total RNA was isolated using the TRIzol reagent and concentration was assessed in a NanoDrop-1000 spectrophotometer (NanoDrop Technologies, Wilmington, DE). RNA integrity was tested with a RNA 6000 Nano Assay in a Bioanalyzer 2100 (Agilent Technologies, Santa Clara, CA). cDNA synthesis of 0.5–1 μg of total RNA was performed using the High-Capacity cDNA Archive Kit (Applied Biosystems). Quantitative analysis of gene expression was performed by real-time PCR in an ABI Prism 7900 Sequence Detection System in Fast Real Time System. We carried out real-time PCR using optimal and pre-designed TaqMan Gene Expression Assays (COX-2 (Hs00153133_m1 and Mm00478374_m1), mPGES-1 (Hs 01115610_m1), mPGES-2 (Hs00228159_m1), cPGES (Hs00832847_gH)), UCP1 (Hs00367969_m1), PRDM16 (Hs00922674_m1), Col1α1 (Hs00164004 and Mm00801666_g1), Col1α2 (Hs01028969_m1 and Mm00483888_m1), TGF-β (Hs00998133_m1 and Mm03023971_m1), TIMP-1 (Hs00171558 and Mm01341361_m1), αSMA (Hs00559403_m1 and Mm00725412_s1), IL-6 (Hs00174122_m1 and Mm00446190_m1) and MCP-1 (Hs00234140_m1 and Mm00441242_m1). β-actin (Hs00969077_m1 and Mm00607939_s1) was used as endogenous control. PCR results were analyzed with the Sequence Detector Software version 2.1 (Applied Biosystems). Relative quantification of gene expression was performed using the comparative Ct method. The amount of target gene normalized to β-actin and relative to a calibrator was determined by the arithmetic equation 2^-ΔΔCt^ described in the comparative Ct method.

### Analysis of protein expression by western blot

Tissue samples were homogenized with 50 mM HEPES, 20 mM β-glycerol phosphate, 2 mM EDTA, 1% Igepal, 10% glycerol, 1 mM MgCl_2_, 1 mM CaCl_2_ and 150 mM NaCl, supplemented with protease inhibitor (Complete Mini) and phosphatase inhibitor (PhosSTOP) cocktails (Roche, Basel, Switzerland). A total of 60 μg protein per tissue were resuspended in SDS-containing Laemmli sample buffer, heated for 5 min at 95°C, and resolved in 15% SDS-PAGE. The transfer was performed in the iBlot Dry Blotting System (Invitrogen) at 20 V for 7 min. The transfer was visualized by Ponceau S solution. Membranes were then soaked for 1 h at room temperature in TBS (20 mM Tris/HCL pH 7.4 and 0.5 M NaCl) containing 0.1% (v/v) Tween 20 (0.1% TBST) and 5% (w/v) non-fat dry milk. The blots were washed three times for 5 min each with 0.1% TBST and subsequently incubated overnight at 4°C with primary anti-phospho-HSL (dilution 1:1000) and anti-total-HSL (dilution 1:1000) (Cell Signaling, Danvers, MA) antibodies in 0.1% TBST containing 5% BSA. After washing the blots three times for 5 min each with 0.1% TBST, the membranes were incubated for 1 hour at room temperature with an HRP-linked donkey anti-rabbit secondary (dilution 1:2000) antibody (Biolegend, San Diego, CA) in 0.1% TBS-T. Bands were visualized using the EZ-ECL chemiluminescence detection Kit (Biological Industries) in a LAS 4000 imaging system (GE Healthcare Life Sciences, San Francisco, CA) and quantified using Image GE ImageQuant TL analysis software.

### Statistical analysis

Statistical significance for the inferred association between target RNAs and biological pathways/processes was assessed by the Fisher’s z-score a measure of the likelihood that the association between a set of genes and a given pathway is not due to random chance. For comparison of 3 groups or conditions, ANOVA was used. For other statistical analysis the Student’s t-test was used. Differences between lean and obese subjects were adjusted for age using univariate analysis based on a linear regression model. The results are expressed as mean ± SEM, and differences were considered significant at *P* < 0.05. The results are expressed as mean ± SEM, and differences were considered significant at *P* < 0.05.

## Results

### Patient characteristics and assessment of adipose tissue fibrosis

The demographic and clinical characteristics of the patients included in the study are shown in S2 Table. Obese individuals were younger and had a greater body weight and body mass index (BMI) compared to non-obese individuals. There were no statistically significant differences in serum aspartate aminotransferase (AST), alanine aminotransferase (ALT), glucose, cholesterol and triglyceride (TAG) levels between the two study cohorts.

**Fig 1:**
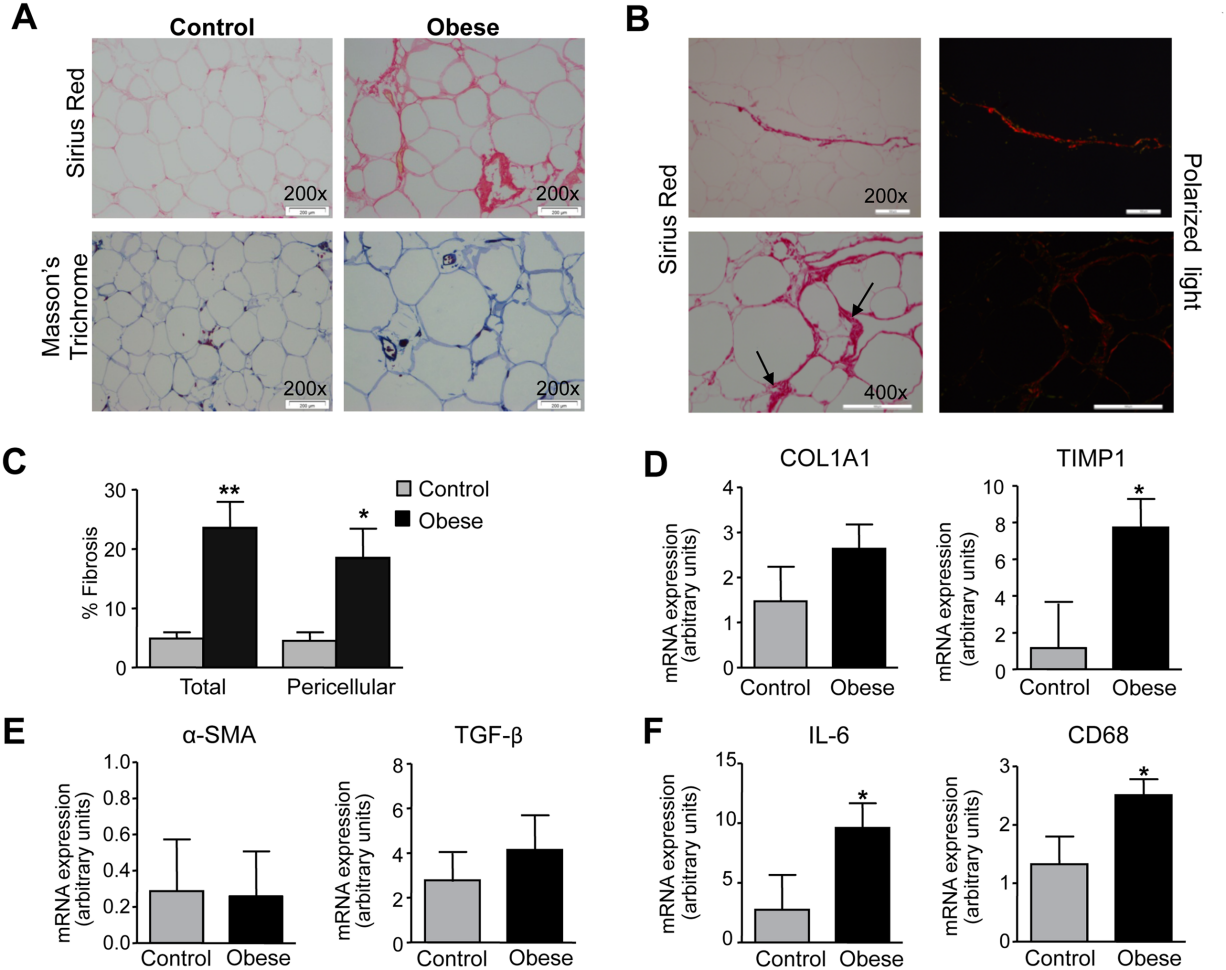
Increased fibrosis and inflammation in obese adipose tissue. **(A)** Representative photomicrographs (×200 magnification; scale bars: 200 μm) of omental white adipose tissue (WAT) sections stained with Sirius Red and Masson’n trichrome from non-obese control individuals (n = 8) and obese patients (n = 8). **(B)** Representative photomicrographs (×200 and x400 magnifications; scale bars: 100 μm) of obese WAT sections stained with Picrosirius Red (left panels) and visualized under polarized light (right panels). Arrows indicate accumulation of fibrosis around adipocytes. **(C)** Total and pericellular fibrosis expressed as percentage of the total cellular membranes in omental WAT from control and obese individuals. **(D,E)** mRNA expression for COLα1, TIMP1, αSMA and TGF-β in WAT from control and obese individuals. **(F)** mRNA expression of inflammatory markers IL-6 and CD68 in WAT. Results are expressed as mean ± SEM. *, P<0.01 and **, P<0.005 versus control.

Histological analysis of WAT sections revealed that in addition to increased adipocyte hypertrophy, obesity was associated with abnormal collagen deposition. Indeed, Sirius Red and Masson’s trichrome staining, which specifically stain collagen fibers, were increased in omental WAT from obese patients as compared to that from non-obese controls **(**Fig 1A**)**. Total as well as pericellular fibrosis were analyzed by visualization of collagen fibers (red, type I collagen; green, type III collagen) under polarized light microscopy **(**Fig 1B**)**. Quantification of total fibrosis (sum of collagen I and III fibers) and pericellular fibrosis is shown in Fig 1C. A statistically significant up-regulation of the fibrosis marker TIMP1 was also seen in WAT from obese individuals, whereas changes in collagen type I alpha 1 (Col1α1), α-smooth muscle actin (αSMA) and TGF-β expression did not reach statistical significance (Fig 1D and 1E). In parallel to exacerbated fibrosis, obese WAT showed significant up-regulation of the inflammatory genes interleukin (IL)-6 and the macrophage marker CD68 (Fig 1F).

### High-throughput analysis of gene expression in adipose tissue

**Fig 2:**
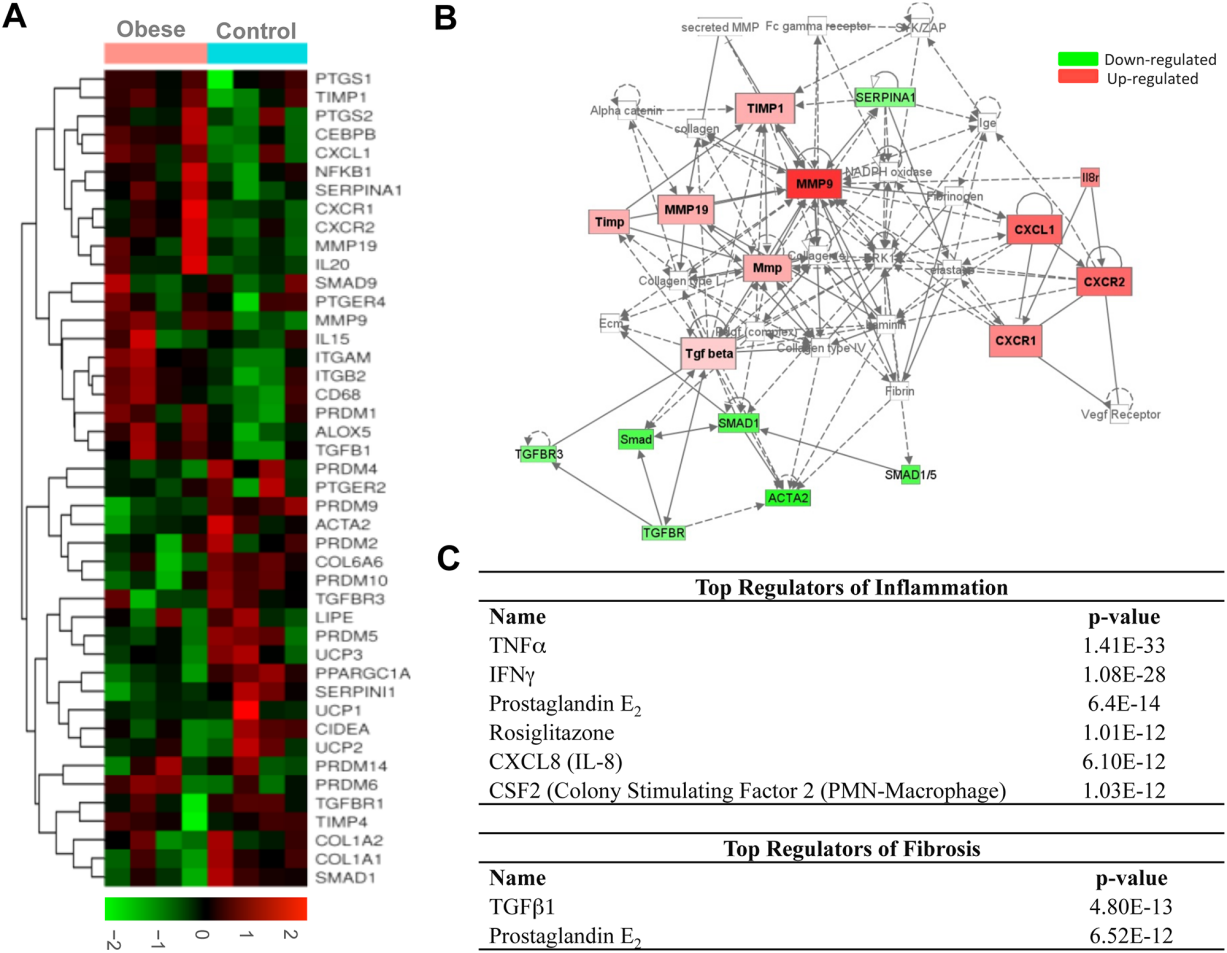
High-throughput transcriptomic analysis of adipose tissue from control and obese individuals. **(A)** Differential gene expression by Affymetrix Human Genome U219 expression arrays in omental adipose tissue from non-obese control individuals (n = 4) and obese patients (n = 4). The results are expressed as a matrix view of gene expression data (heat map) in which rows represent genes and columns represent hybridized samples. The intensity of each colour denotes the standardized ratio between each value and the average expression of each gene across all samples. Red pixels correspond to an increased abundance of mRNA in the samples indicated, whereas green pixels indicate decreased mRNA levels. **(B)** Integrative IPA of transcriptomic data. Genes significantly down-regulated are indicated in green; those that were significantly up-regulated are indicated in red. **(C)** Cascade of top regulators of inflammation and fibrosis detected by IPA software.

In order to investigate changes in the profile of gene expression, we performed high-throughput transcriptome analysis in omental WAT from obese and non-obese individuals. This analysis identified a group of genes associated with the inflammation-fibrosis axis that was differentially modulated in obesity **(**Fig 2A**)**. Among the genes involved in fibrosis, we identified changes in members of the collagen synthesis (COL6A6 and COL1A1), metalloproteases (MMP9 and MMP19), tissue inhibitors of metalloproteases (TIMP1) and TGF-β signaling cascade (TGFB1 and SMAD1) and αSMA (ACTA2) (Fig 2A). Among the inflammatory genes, we found significant up-regulation of members of the cyclooxygenase (COX) and lipoxygenase (LOX) pathways (PTGS2 (COX-2), PTGER4 (PGE_2_ receptor 4) and ALOX5 (5-LOX)); integrins (ITGAM and ITGB2); cytokines (IL20 and IL15) as well as IL-6, IL-1β and IL-10 (not shown in the heat-map); chemokines (i.e. CXCL1 (GROα), CXCR1, and CXCR2 (IL-8 receptors)) as well as MCP-1 (not shown in the heat-map) and CD68. Over-expression of the inflammatory adipokines resistin and leptin was also seen (data not shown). On the other hand, the transcriptome analysis identified the up-regulation of genes involved in adipogenesis (CEBPB) and a significant down-regulation of a cluster of genes involved in WAT browning (i.e. PRDM4, PDRM9, PDRM10 and PPARGC1A). By means of functional Ingenuity Pathway Analysis (IPA), we confirmed the presence of a strong association between changes in genes involved in inflammation and fibrosis in WAT from obese patients (Fig 2B). Moreover, IPA analysis identified a number of pathways related to inflammation and fibrosis as the top canonical pathways differentially regulated in WAT from obese patients (S3 Table). Finally, IPA analysis identified PGE_2_ as a common top regulator of the inflammatory and fibrogenic processes in obese WAT (Fig 2C).

### Assessment of PG levels by LC-ESI-MS/MS

**Fig 3:**
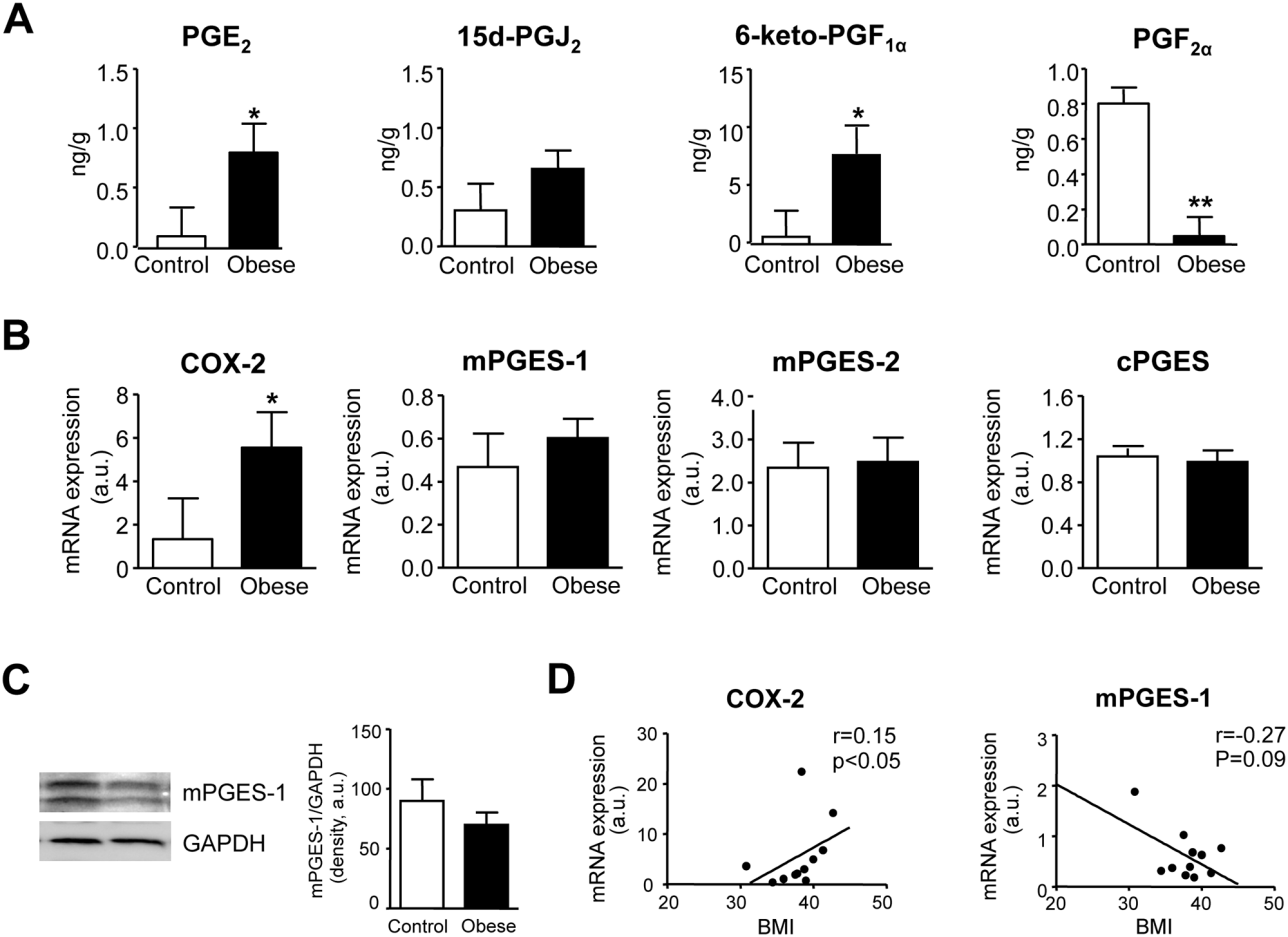
Targeted lipidomic analysis of COX products in adipose tissue from control and obese individuals. **(A)** Levels of PGE_2_, 15d-PGJ_2_, 6-keto-PGF_1α_ and PGF_2α_ in adipose tissue from non-obese control individuals (n = 6) and obese patients (n = 7) were assessed by LC-ESI-MS/MS. **(B)** COX-2, mPGES-1, mPGES-2 and cPGES expression in omental adipose tissue from control (n = 7) and obese (n = 11) individuals. **(C)** Representative Western blot of mPGES-1 protein expression in omental adipose tissue. **(D)** Correlation between the expression of COX-2 and mPGES-1 in omental adipose tissue and the body mass index (BMI). The results are expressed as mean ± SEM. *, P<0.05 and **, P<0.01 versus control.

We next assessed PG levels in omental WAT from obese and non-obese individuals by targeted lipidomic LC-ESI-MS/MS analysis. As shown in Fig 3A, significantly higher levels of PGE_2_ and 6-keto-PGF_1α_, the inactive PGI_2_ metabolite, were detected in obese WAT compared to samples from non-obese controls. In contrast, PGF_2α_ was significantly reduced in obese adipose tissue (Fig 3A). Levels of PGD_2_ (data not shown) and its adipogenic metabolite, 15d-PGJ_2_ (Fig 3A) were not different between the two groups. TXB_2_, PGJ_2_ and PGH_2_ levels were undetectable (data not shown). Increased PGE_2_ levels occurred in the context of increased COX-2 expression without changes in the terminal prostaglandin E synthases (i.e. mPGES-1, mPGES-2 and cPGES) (Fig 3B). Absence of changes in the expression of mPGES-1 was confirmed at the protein level by western blot analysis (Fig 3C). This finding is consistent with the down-regulation of mPGES-1 in adipose tissue from high-fat diet induced obese mice [20]. Interestingly, the expression of COX-2, but not mPGES-1, increased in a BMI-dependent manner and closely correlated with the degree of obesity (Fig 3D).

### Effects of PGE_2_ on WAT fibrosis

**Fig 4:**
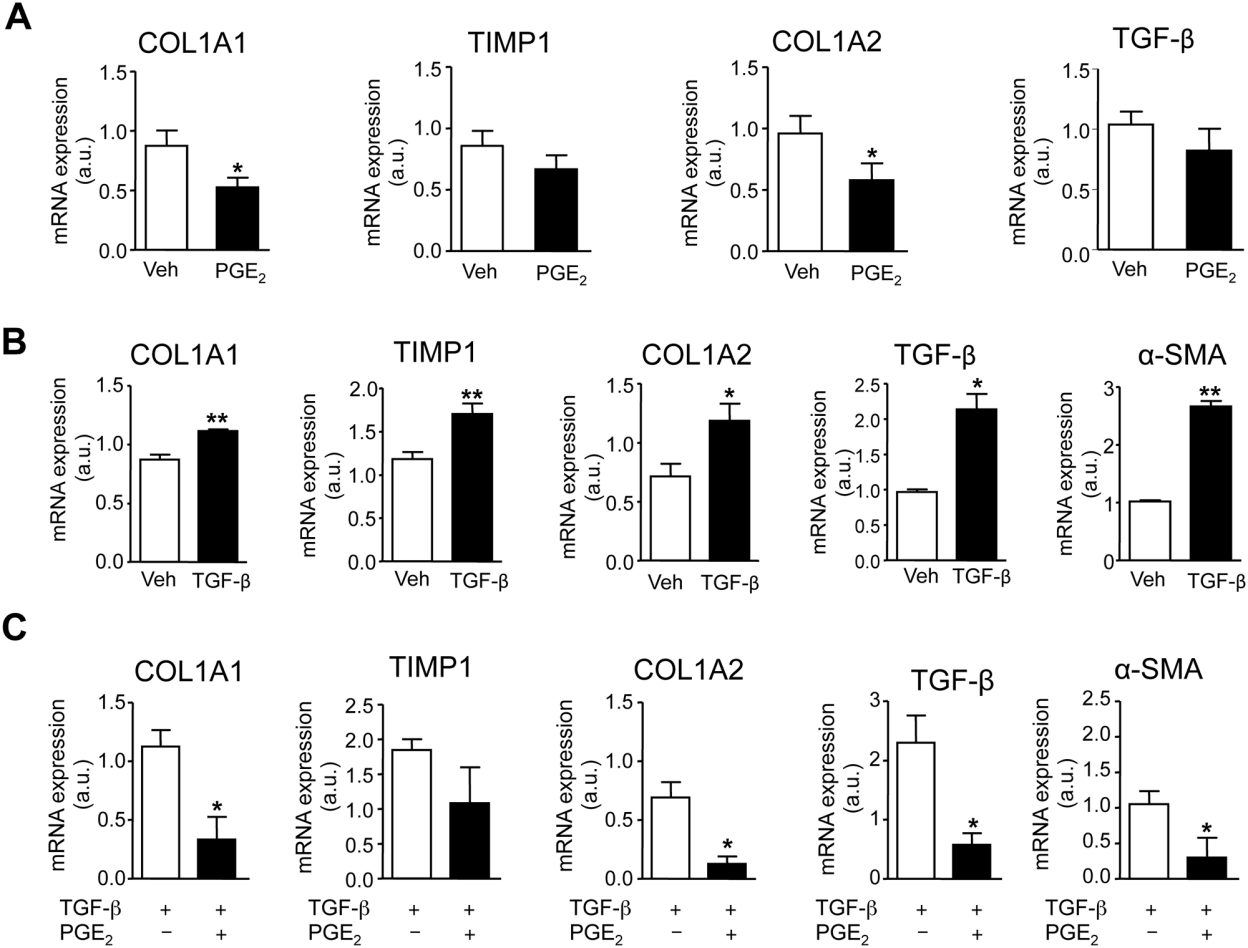
Effects of PGE_2_ on adipose tissue remodeling. **(A)** mRNA expression for COL1α1, TIMP1, COL1α2 and TGF-β in obese fat pads incubated with vehicle (Veh) or PGE_2_ (1 μM). **(B)** mRNA expression for COL1α1, TIMP1, COL1α2, TGF-β and αSMA in differentiated 3T3-L1 adipocytes growing in the presence of Veh, (0.19% DMSO) or TGF-β (10 ng/ml). **(C)** mRNA expression for COL1α1, TIMP1, COL1α2, TGF-β and αSMA in 3T3-L1 adipocytes growing in the presence of TGF-β (10 ng/ml) and incubated with vehicle (Veh) or PGE_2_ (0.1 μM). The results are expressed as mean ± SEM. *, P<0.05 and **, P<0.01 versus Veh.

We then evaluated the effects of PGE_2_ on the fibrotic process in WAT explants from obese individuals. As shown in Fig 4A, incubation of fat explants with PGE_2_ significantly reduced the expression of the fibrogenic genes Col1α1 and Col1α2 (Fig 4A). Changes in TIMP1 and TGF-β did not reach statistical significance (Fig 4A). Expression of αSMA was not detected in un-stimulated adipose tissue explants (data not shown). To further investigate the effects of PGE_2_ on adipose tissue remodeling, we performed *in vitro* experiments in mature adipocytes. Since the surgical pieces of fat collected in the surgical room yielded an insufficient number of pre-adipocytes, the *in vitro* experiments were performed in adipocyte-differentiated 3T3-L1 cells, which allowed us enough biological material for performing extensive *in vitro* studies. As shown in Fig 4B, 3T3 adipocytes growing in the presence of the pro-fibrogenic cytokine TGF-β showed a significant up-regulation in Col1α1, TIMP1, Col1α2, TGF-β and αSMA. Under TGF-β stimulation PGE_2_ produced a significant down-regulation of the fibrogenic genes Col1α1, Col1α2, TGF-β and αSMA (Fig 4C).

### Effects of PGE_2_ on WAT inflammation

**Fig 5:**
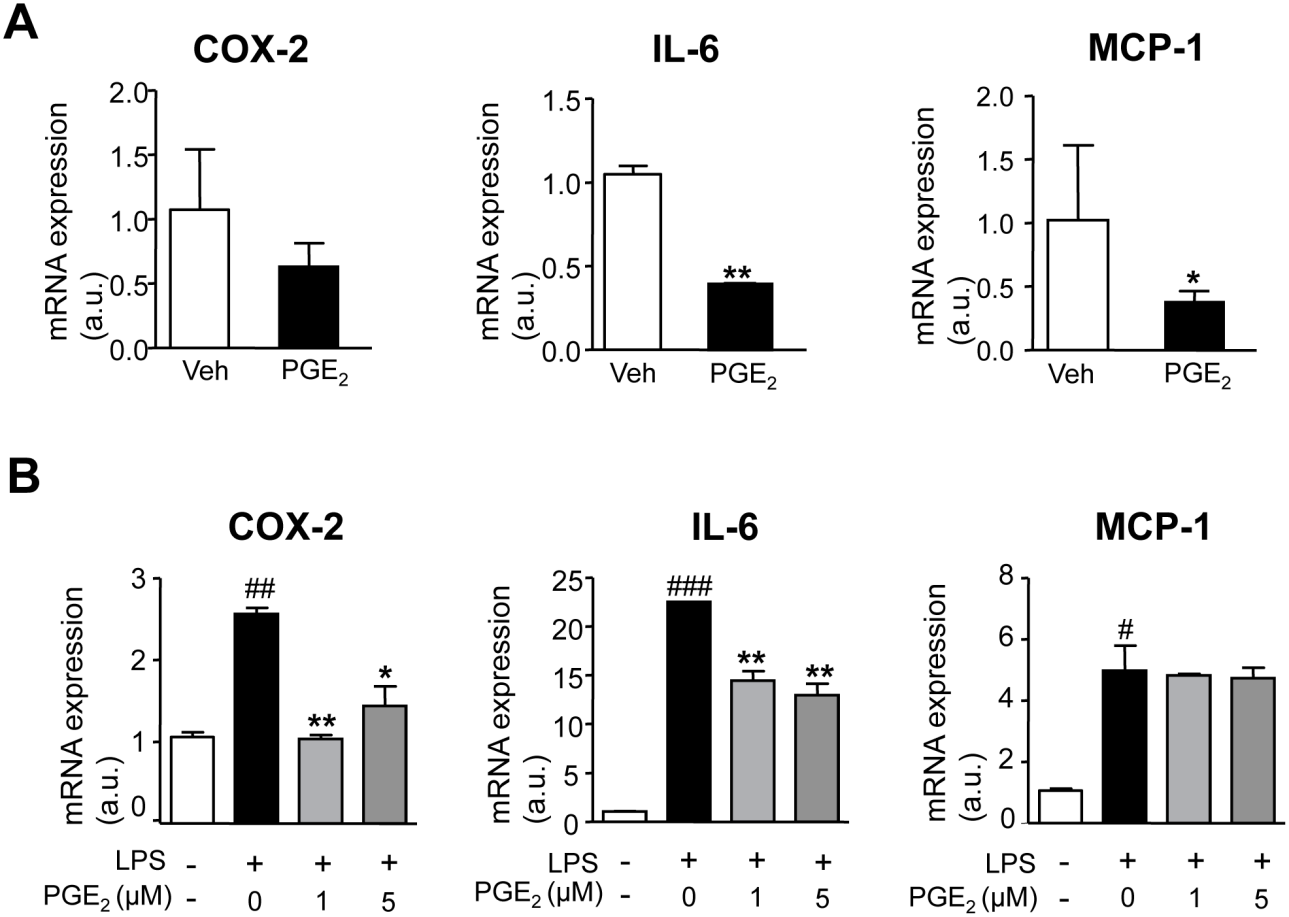
Anti-inflammatory effects of PGE_2_ on adipose tissue. **(A)** mRNA expression for COX-2, IL-6 and MCP-1 in human obese fat pads incubated with vehicle (Veh) or PGE_2_ (1 μM). **(B)** COX-2, IL-6 and MCP-1 expression in differentiated 3T3-L1 adipocytes growing in the presence of LPS (100 ng/ml) and incubated with increasing concentrations of PGE_2_ (0, 1 and 5 μM). The results are expressed as mean ± SEM. #, P<0.05, ##, P<0.01 and ###, P<0.001 for LPS versus untreated cells. *, P<0.05 and **, P<0.01 versus Veh or LPS.

We also studied the effects of PGE_2_ on the inflammatory process in obese WAT. As shown in Fig 5A, the addition of exogenous PGE_2_ to fat explants resulted in a reduced expression of the inflammatory cytokines IL-6 and MCP-1. Consistent with this finding, PGE_2_ significantly prevented the up-regulated expression of COX-2 and IL-6 in 3T3 adipocytes exposed to LPS (Fig 5B). The anti-inflammatory actions of PGE_2_ were subsequently confirmed by microarray analysis of human pre-adipocytes incubated with this eicosanoid (S1 Fig).

### Effects of PGE_2_ on WAT browning

**Fig 6:**
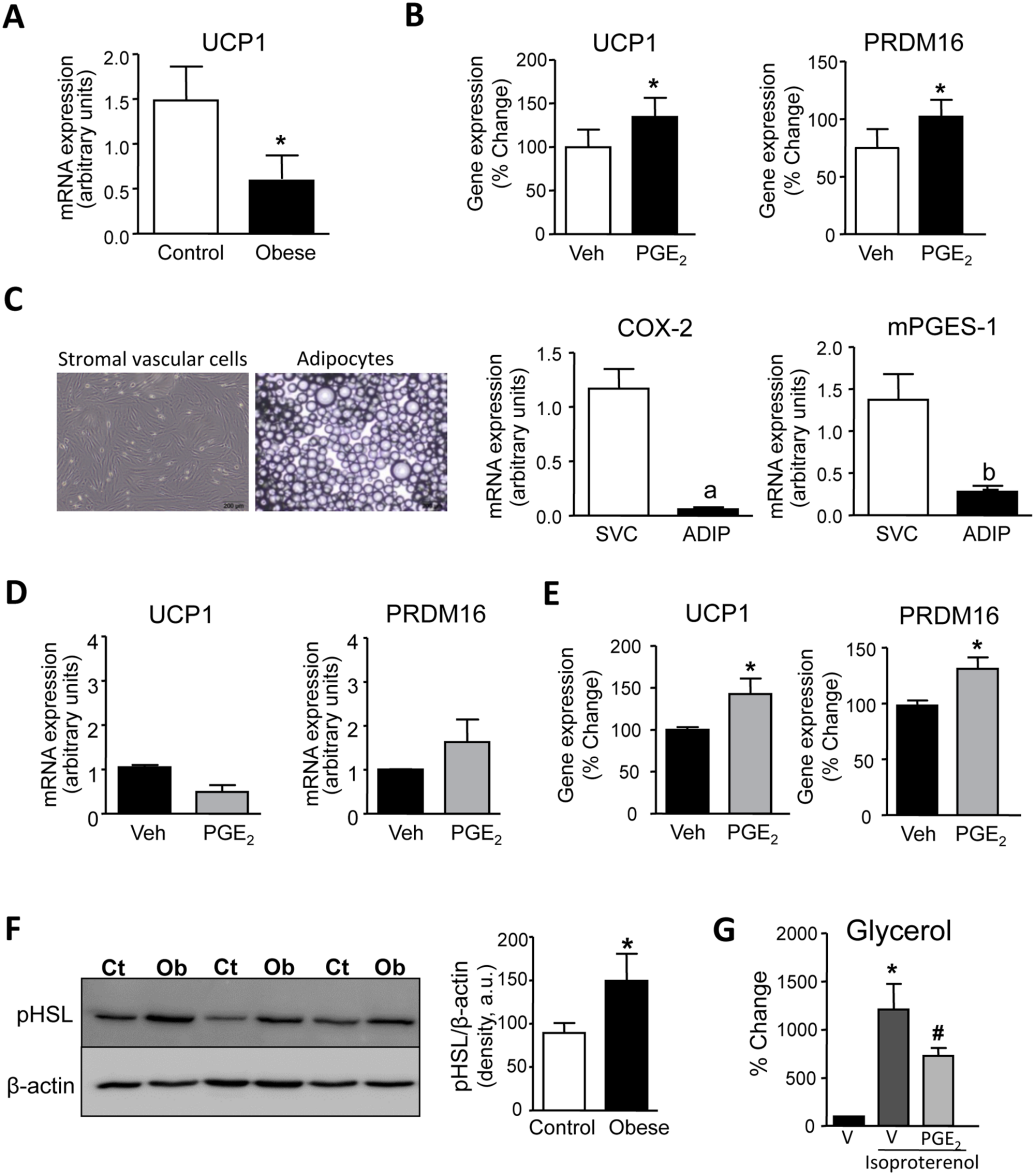
Effects of PGE_2_ on beige/brite differentiation. **(A)** Adipose tissue expression of UCP1 in adipose tissue from non-obese control individuals (n = 10) and obese patients (n = 11). **(B)** Expression of UCP1 and PRDM16 in obese fat pads incubated with vehicle (Veh, 0.19% DMSO) or PGE_2_ (1 μM) for 24 hours. **(C)** Representative photomicrographs of stromal vascular cell (SVC) and adipocyte cultures isolated from adipose tissue of obese patients (x200 magnification; scale bars; 200 μm). ***Right panel***: Expression of COX-2 and mPGES-1 in SVC and adipocytes from obese adipose tissue. **D)** Expression of UCP1 and PRDM16 in differentiated pre-adipocytes isolated from obese patients growing in the presence of Veh (0.19% DMSO) or PGE_2_ (1 μM). **(E)** Expression of UCP1 and PRDM16 in adipocytes isolated from obese adipose tissue and incubated with Veh (0.19% DMSO) or PGE_2_ (1 μM) for 3 h. **(F)** Representative western blot of phosphorylated HSL (pHSL) protein expression in adipose tissue from non-obese control individuals (CT) (n = 7) and obese patients (Ob) (n = 8). **(G)** Glycerol levels released by adipocytes incubated with vehicle (V) (0.19% DMSO) or isoproterenol (1 μM) in the presence of V or PGE_2_ (1 μM) for 1.5 h. The results are expressed as mean ± SEM. *, P<0.05 versus control or Veh; a, P<0.05 and b P<0.01 versus SVC. #, P<0.05 versus isoproterenol-treated adipocytes.

Thereafter we investigated the effects of PGE_2_ on the browning of human omental WAT. Previous studies from our laboratory in an experimental model of high-fat diet-induced obesity have demonstrated that PGE_2_ stimulates the differentiation of pre-adipocytes of WAT origin into beige/brite adipocytes [16]. In the present study, the expression of the brown marker UCP1 was found to be significantly reduced in omental WAT of obese patients (Fig 6A). The exogenous addition of PGE_2_ to human WAT explants significantly up-regulated UCP1 and PRDM16 expression (Fig 6B). To investigate which adipose tissue fraction is involved in the PGE_2_ actions, we next isolated adipocytes and SVC and analyzed the relative expression of COX-2 and mPGES-1. As shown in Fig 6C, the expression of these PGE_2_-generating enzymes was mostly ascribed to the SVC fraction, which is highly populated by pre-adipocytes. Accordingly, we isolated pre-adipocytes from human omental WAT and incubated these cells with PGE_2_. As shown in Fig 6D, PGE_2_ was not able to induce a consistent induction of UCP1 and PRDM16 in human pre-adipocytes during the process of differentiation into mature adipocytes. In contrast, PGE_2_ significantly increased the expression of UCP1 and PRDM16 in adipocytes (Fig 6E), suggesting that the browning of human omental WAT could be the consequence of the trans-differentiation of mature adipocytes.

### Effects of PGE_2_ on WAT lipolysis

Finally, we explored the effects of PGE_2_ on WAT lipolysis. As expected, human omental WAT showed remarkable phosphorylation of the lipolytic enzyme hormone-sensitive lipase (HSL) (Fig 6F). Incubation of human adipocytes with the lipolytic compound isoproterenol resulted in enhanced adipocyte lipolysis, an effect that was significantly decreased by PGE_2_ (Fig 6G).

## Discussion

The results of the current investigation demonstrate the presence of increased levels of PGE_2_ in the adipose tissue of obese subjects as compared to lean individuals. The increased levels of this prostanoid were not related to an overexpression of mPGES-1, the main PGE synthase in adipose tissue cells [21], but rather with COX-2, which expression was found remarkably up-regulated in obese adipose tissue. In addition, the current study provides evidence that exogenous addition of PGE_2_ exerts multiple effects on the homeostatic control of obese adipose tissue. Specifically, our results identified PGE_2_ as a potent regulator of the pathologic processes leading to uncontrolled inflammation and fibrosis and impaired adaptive thermogenesis and lipolysis in white adipose tissue during obese conditions.

An interesting finding of our study was the observation that PGE_2_ had the ability to regulate WAT fibrosis. Fibrosis is a hallmark of metabolically dysfunctional WAT in which adipocytes are surrounded by a network of extracellular matrix proteins [10,12]. The exaggerated accumulation of extracellular matrix reduces the plasticity of adipose tissue leading to adipocyte dysfunction [12]. Indeed, total and pericellular fibrosis as determined by a combination of techniques including Sirius Red and Masson’s trichrome staining as well as visualization of collagen fibers (red, type I collagen; green, type III collagen) under polarized light microscopy, clearly indicated the presence of increased fibrosis in omental WAT from obese individuals. In this tissue, PGE_2_ down-regulated the expression of fibrosis-inducing genes in human fat explants from obese individuals as well as the fibrogenic response of differentiated adipocytes to the fibrogenic actions of TGF-β, a cytokine that plays a pivotal role in fibrosis by regulating matrix synthesis and deposition [22]. These findings are novel because, as far as we know, this is the first study assessing the effects of PGE_2_ on WAT fibrosis. Our findings are consistent with those previously reported for PGE_2_ in other tissues and organs. In this regard, PGE_2_ has been reported to exert antifibrotic effects in the lung by inhibiting multiple fibroblast functions as well as fibroblast survival [23]. Similar findings have been reported in the liver where PGE_2_ inhibits TGF-β-mediated induction of collagenα1(I) and delays the mitogenic effect of PDGF-BB in hepatic stellate cells [23,24].

A salient feature of our study was the observation that PGE_2_ exerts anti-inflammatory actions in obese adipose tissue. Indeed, PGE_2_ inhibited the expression of inflammatory genes in obese fat explants and reduced the inflammatory response of differentiated adipocytes challenged with the bacterial product LPS. While contributing to the initiation of inflammation by promoting vasodilation, pain and fever, PGE_2_ has been demonstrated to suppress the activation of immune cells, mainly macrophages [25–28]. This eicosanoid appears to limit nonspecific inflammation as part of immune suppression associated with chronic inflammation. In particular, PGE_2_ mediates suppression of type 1 immunity especially at later stages of immune responses [25,26]. The ability of PGE_2_ to promote tissue host self-preservation has been documented to be mediated by the increase in the levels of the intracellular second messenger cAMP [28]. Alternatively, PGE_2_ has the ability to switch eicosanoid biosynthesis from predominantly inflammatory mediators (such as LTB_4_) to pro-resolution factors (such as LXA_4_) able to stop unremitting inflammation [28]. Although it has recently been suggested that adipocyte inflammation is essential for healthy WAT expansion and remodeling [29], a wealth of evidence indicate adipose tissue inflammation has a fundamental negative impact on metabolism leading to comorbidities associated with obesity, including insulin resistance and non-alcoholic fatty liver disease [11]. Therefore, our observation that PGE_2_ counter-regulates inflammation in obese WAT highlights the potential compensatory actions of this eicosanoid in inflamed WAT.

Our study also provides evidence that PGE_2_ modulates adaptive thermogenesis in omental WAT from obese individuals. In this regard, exogenous addition of PGE_2_ induced the expression of UCP1, a gene that plays a pivotal role in the browning of omental WAT. Previous work from our laboratory has demonstrated that exposure of pre-adipocytes of WAT origin to PGE_2_ results in a browning effect during the adipocyte differentiation process [16]. This seminal discovery was performed in a murine model of obesity and in the current study we translated this finding to humans, demonstrating that PGE_2_ increases brown markers in omental WAT from obese patients. However, there is an important difference between the human and murine results. In humans, PGE_2_ induced UCP1 expression in mature adipocytes but not in pre-adipocytes, whereas in mice, the browning was exclusively ascribed to preadipocytes. Together, these data suggest that the browning of human obese WAT can implicate the trans-differentiation of mature white adipocytes into beige/brite cells. Understanding the mechanisms underlying adipocyte transdifferentiation is likely to open new avenues for the design of novel strategies for the treatment of obesity and associated metabolic disorders [30].

Finally, PGE_2_ exerted anti-lipolytic actions on omental WAT, as estimated by a reduction in the activity of HSL, the most important lipolytic enzyme in WAT. This finding confirms the anti-lipolytic effects of PGE_2_ in humans originally reported in rat WAT [31]. Since augmented HSL activity in obese omental WAT results in increased release of non-essential fatty acids into the circulation and impaired β-cell function, insulin resistance and fatty liver disease [32], PGE_2_ can also be postulated as a key regulator of systemic metabolism.

In summary, the present study demonstrates that PGE_2_ exerts pleiotropic effects on human obese WAT, counter-regulating the unbalanced activity of the inflammation-fibrosis axis, adaptive thermogenesis and lipolysis. The current investigation was performed in omental WAT samples from patients with obesity undergoing laparoscopic bariatric surgery, and therefore our findings are relevant in terms of human obesity.

## Supporting Information

S1 Fig Fold decrease changes with respect to vehicle of genes regulating the inflammatory response in human pre-adipocytes exposed to PGE_2_ (1 μM, 3 h). (PDF)

S1 Table LC-MS/MS conditions for each lipid mediator. (DOCX)

S2 Table Baseline demographic and clinical characteristics of non-obese individuals and obese patients included in the study. (DOCX)

S3 Table Top canonical pathways modified in omental adipose tissue from obese patients compared to lean individuals. (DOCX)
