# Supplementary material for: Prostaglandin E2 Exerts Multiple Regulatory Actions on Human Obese Adipose Tissue Remodeling, Inflammation, Adaptive Thermogenesis and Lipolysis
Source: PLoS One. 2016 Apr 28;11(4):e0153751. doi: 10.1371/journal.pone.0153751 (PMC4849638; doi:10.1371/journal.pone.0153751)

S1 Fig: Fold decrease changes with respect to vehicle of genes regulating the inflammatory response in human pre-adipocytes exposed to PGE_2 (1 µM, 3 h).

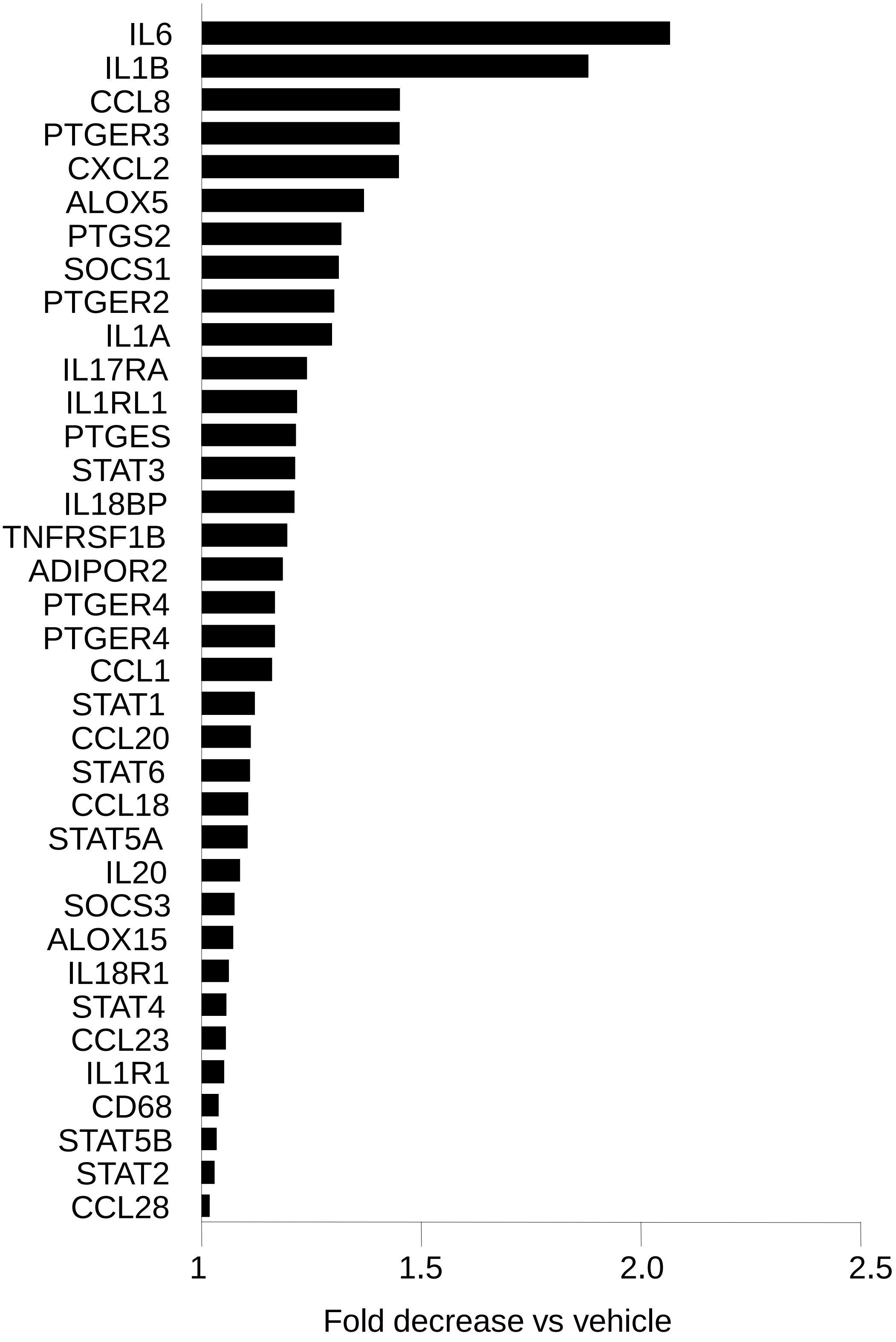

Supplement: S1 Fig — (PDF) [file pone.0153751.s001.pdf]
