# Supplementary material for: Prostaglandin E2 Exerts Multiple Regulatory Actions on Human Obese Adipose Tissue Remodeling, Inflammation, Adaptive Thermogenesis and Lipolysis
Source: PLoS One. 2016 Apr 28;11(4):e0153751. doi: 10.1371/journal.pone.0153751 (PMC4849638; doi:10.1371/journal.pone.0153751)
Supplement: S1 Table — (DOCX) [file pone.0153751.s002.docx]

**S1 Table**. LC-MS/MS conditions for each lipid mediator.

| **Compound Name** | **Transition** | **Fragmentor [V]** | **CE** | **Cell Accelerator [V]** | **Retention Time ^1^ [min]** |
| --- | --- | --- | --- | --- | --- |
| 6-keto-PGF_1α_ | 369.2→163 | 130 | 24 | 8 | 4.83 |
| TXB_2_ | 369.2→195 | 100 | 9 | 1 | 5.18 |
| PGF_2α_ | 353.2→309 | 130 | 13 | 3 | 5.37 |
| PGE_2_ | 351.3→315 | 110 | 4 | 5 | 5.54 |
| PGD_2_ | 351.2→271 | 80 | 9 | 8 | 5.69 |
| PGH_2_ | 351.2→315 | 110 | 4 | 2 | 6.29 |
| PGJ_2_ | 333.3→271 | 110 | 11 | 4 | 6.58 |
| 15-deoxy-Δ12,14-PGJ_2_ | 315.2→271 | 130 | 9 | 1 | 9.6 |

**^1^**Retention times refer to the Kinetex C-18, 2.1 x 150 mm, 2.6 µm column using a solvent system of aqueous formic acid (0.1%) and acetonitrile. The elution gradient was started with 5 % acetonitrile, which was increased within 0.5 minutes to 55%, 14.5 minutes to 69 %, 14.6 minutes to 95 % and held there for 5.4 minutes. The flow rate was set at 0.3 mL/min, the injection volume was 7.5 µL.CE-colision energy
