# Supplementary material for: Prostaglandin E2 Exerts Multiple Regulatory Actions on Human Obese Adipose Tissue Remodeling, Inflammation, Adaptive Thermogenesis and Lipolysis
Source: PLoS One. 2016 Apr 28;11(4):e0153751. doi: 10.1371/journal.pone.0153751 (PMC4849638; doi:10.1371/journal.pone.0153751)
Supplement: S2 Table — (DOCX) [file pone.0153751.s003.docx]

**S2 Table.** Baseline demographic and clinical characteristics of non-obese individuals and obese patients included in the study.

| **Values** | **Non-Obese**  **(n=10)** | **Obese**  **(n=12)** | **P value** |
| --- | --- | --- | --- |
| Age | 62±8 | 53±10 | <0.05 |
| Body Weight (kg) | 70±12 | 106±17 | <0.001 |
| BMI (kg/m^2^) | 26.4±3.2 | 37.9±3.4 | <0.001 |
| Male/Female | 3/6 | 5/7 |  |
| AST (UI/L) | 22.5±12.8 | 21.9±7.1 | ns |
| ALT (UI/L) | 27.7±20.6 | 25.4±8 | ns |
| Glucose (mg/dL) | 116±51.8 | 125.8±68.3 | ns |
| Cholesterol (mg/dL) | 162.3±57.2 | 173.9±26.7 | ns |
| TAG (mg/dL) | 129.5±84.4 | 126.5±51.9 | ns |

BMI: body mass index, AST: aspartate aminotransferase, ALT: alanine aminotranferase; TAG: triglycerides; ns: not significant
