# Supplementary material for: Prostaglandin E2 Exerts Multiple Regulatory Actions on Human Obese Adipose Tissue Remodeling, Inflammation, Adaptive Thermogenesis and Lipolysis
Source: PLoS One. 2016 Apr 28;11(4):e0153751. doi: 10.1371/journal.pone.0153751 (PMC4849638; doi:10.1371/journal.pone.0153751)
Supplement: S3 Table — (DOCX) [file pone.0153751.s004.docx]

**S3 Table.** Top canonical pathways modified in omental adipose tissue from obese patients compared to lean individuals.

| Top Canonical Pathways | |
| --- | --- |
| Pathway | **p-value** |
| Complement system | 2.7E-10 |
| Granulocyte Adhesion and Diapedesis | 5.27E-09 |
| LXR/RXR Activation | 9.1E-08 |
| Fcγ Receptor-mediater Phagocytosis in Macrophages and Monocytes | 3.37E-07 |
| Atherosclerosis Signaling | 8.92E-07 |
| Agranulocyte Adhesion and Diapedesis | 3.1 E-06 |
| Production of Nitric Oxide Oxygen Species in Macrophages | 5.01E-05 |
| IL-10 Signaling | 1.2E-04 |
| IL-8 Signaling | 3.1E-04 |
| Fibrosis/Stellate Cell Activation | 4.2E-04 |
| IL-12 Signaling and Production in Macrophages | 1.69E-03 |
| Eicosanoid Signaling | 3.2E-03 |
| IL-6 signaling | 3.1E-03 |
| Type II Diabetes Mellitus Signaling | 0.034 |
| Adipogenesis pathway | 0.019 |
| LPS-stimulated MAPK | 0.031 |
| STAT 3 Pathway | 0.031 |
| Leukotrine Biosynthesis | 0.025 |
| Fatty Biosynthesis I | 0.03 |
| CD28 Signaling in T helper Cells | 0.012 |

P-value (calculated by Fisher´s exact test) determines the probability that the association between the genes in the dataset and the canonical pathway is explained by chance alone
